# Relative Measures of Association for Binary Outcomes: Challenges and Recommendations for the Global Health Researcher

**DOI:** 10.5334/aogh.2581

**Published:** 2019-11-20

**Authors:** John A. Gallis, Elizabeth L. Turner

**Affiliations:** 1 Duke Global Health Institute, Duke University, Durham, North Carolina, US; 2 Department of Biostatistics and Bioinformatics, Duke University School of Medicine, Durham, North Carolina, US

## Abstract

**Background:**

Binary outcomes—which have two distinct levels (e.g., disease yes/no)—are commonly collected in global health research. The relative association of an exposure (e.g., a treatment) and such an outcome can be quantified using a ratio measure such as a risk ratio or an odds ratio. Although the odds ratio is more frequently reported than the risk ratio, many researchers, policymakers, and the general public frequently interpret it as a risk ratio. This is particularly problematic when the outcome is common because the magnitude of association is larger on the odds ratio scale than the risk ratio scale. Some recently published global health studies included misinterpretation of the odds ratio, which we hypothesize is because statistical methods for risk ratio estimation are not well known in the global health research community.

**Objectives:**

To compare and contrast available statistical methods to estimate relative measures of association for binary outcomes and to provide recommendations regarding their use.

**Methods:**

Logistic regression for odds ratios and four approaches for risk ratios: two direct regression approaches (modified log-Poisson and log-binomial) and two indirect methods (standardization and substitution) based on logistic regression.

**Findings:**

Illustrative examples demonstrate that misinterpretation of the odds ratio remains a common issue in global health research. Among the four methods presented for estimation of risk ratios, the modified log-Poisson approach is generally preferred because it has the best numerical performance and it is as easy to implement as is logistic regression for odds ratio estimation.

**Conclusions:**

We conclude that, when study design allows, studies with binary outcomes should preferably report risk ratios to measure relative association.

## Introduction

Binary outcomes—which have two distinct levels (e.g., disease yes/no)—are commonly measured in global health research. Examples include depression status [1], disease status [2], and mortality [3], among others. These binary outcomes may either be true “yes or no” variables (e.g., mortality) or be created from an underlying continuous variable (e.g., when depression status is determined by dichotomizing a psychological scale). For example, one recent article created a binary HIV-related knowledge variable by dichotomizing a total HIV-related knowledge score at the median [4].

The relative association of an exposure and binary outcome can be quantified through the use of a ratio measure such as a risk ratio or odds ratio. The risk ratio is defined as the risk of the outcome in the exposed group over the risk of outcome in the unexposed group, where an exposure could be a treatment (intervention) assignment or some other binary predictor (e.g., obesity yes/no). For example, using data from a randomized controlled trial (RCT) of an intervention to increase the proportion of febrile individuals testing for malaria [5], the estimated “risk” of testing for malaria is higher in treatment than control (Table 1; risk ratio = 1.45). The odds ratio is defined as the odds of the outcome in the exposed group over the odds of the outcome in the unexposed group, where the odds of the outcome in a group is the proportion with the outcome over the proportion without the outcome. The odds ratio for this example is 2.7, which is larger than the risk ratio.

**Table 1:** Example of two relative measures of association, adapted from results of a randomized controlled trial in febrile individuals published in *BMJ Global Health*.^a^

Exposure group	Outcome^b^			Relative Measure of Associationc	

	Tested for malaria	Did not test for malaria	“Risk” of malaria testing	Risk Ratio	Odds Ratio

Intervention	76	27	76/103 = 73.8%	73.8/51.0 = 1.45	\frac{{76}}{{27}}/\frac{{51}}{{49}} = 2.7
Control	51	49	51/100 = 51.0%		

^a^ RCT by O’Meara et al. (2016) [5] was a 2 × 2 factorial design of two interventions for febrile individuals. Here we have adapted the example to focus on one of those interventions, namely a subsidy for a rapid diagnostic test, where “intervention” denotes the group that received the subsidy and “control” denotes the group that did not receive the subsidy. Specifically, we have extracted outcome data from Table 2 of O’Meara et al. (2016) [5] for the two groups which did not receive the second intervention. ^b^ Row counts correspond to the number of participants with each level of the outcome within each exposure group. ^c^ For intervention group vs. control group, where we note that O’Meara et al. (2016) [5] reported neither of these results in their Table 2 because they instead reported absolute measures of effect.

The odds ratio (OR) is the only valid measure of relative association in traditional case-control studies, namely cumulative case-control studies, because the sampling of controls (e.g., survivor sampling) does not provide a valid estimate of the risk of exposure in the source population [6]. But for studies that use sampling that is dependent on the exposure of interest—including cohort and cross-sectional studies, and randomized controlled trials—the risk ratio (RR) is a valid alternative measure of relative association. Yet, in many of these studies, the OR is the only relative measure of associated reported [7]. This popularity is likely because it is straightforward to implement the logistic regression approach that is typically used to estimate ORs.

Despite its widespread use, the OR is frequently misinterpreted as an RR by researchers, journalists, policymakers, and the general public [8]. As shown by the example above, interpreting the OR of 2.7 as an RR would considerably overstate the impact of the intervention evaluated in this RCT. Such a large difference in magnitude between the two relative measures of associations arises here because the reference (control) arm risk (51%) shows that malaria testing is a common outcome in the study setting. In contrast, in situations where the reference risk is not large because the outcome is not common (e.g., <10%), the odds ratio would approximate the risk ratio and therefore the potential for misinterpretation is greatly reduced.

The purpose of the current paper is to provide researchers with the tools to be able to obtain appropriate and interpretable measures of relative association in studies with binary outcomes. To do so, we compare and contrast the risk ratio and odds ratio, provide examples of the misinterpretation of odds ratios from the recent global health literature, describe methods for obtaining risk ratios in analyses of binary outcomes, and make recommendations for selecting the most appropriate analysis. In addition, we briefly discuss the merits of including an absolute measure of association along with a relative measure. Our goal is to assist global health researchers in making informed decisions about when to report the odds ratio or the risk ratio to measure relative association.

## Relative Measures of Association

### Motivating Example

In the introduction, we presented an example from O’Meara et al. who reported the results of a 2 × 2 factorial RCT, examining the independent and combined effects of two different subsidy interventions (subsidies for rapid diagnostic tests and subsidies for malaria treatment) on the proportion of febrile individuals testing for malaria, a binary outcome [5]. For simplicity, we considered only one of the interventions, namely subsidies for rapid diagnostic tests (RDTs), ignoring the fact that a second intervention was evaluated in the study, and reproduced the reported outcome data from Table 2 of O’Meara et al. [5] (Table 1). The probability (“risk”) of testing for malaria in the RDT subsidy arm is 73.8%, whereas this probability is 51% in the no subsidy (control) arm. Thus, as noted above, the estimated RR is 1.45, and the estimated OR is 2.7. That is, the RDT subsidy is associated with 1.45 times the “risk”, or 2.7 times the odds of malaria testing when compared with no subsidy.

**Table 2:** Unadjusted measures of relative association from three articles in the global health literature.

	Exposure	Outcome	Unexposed group outcome proportion	Risk Ratio^a^	Odds Ratio^b^	Magnitude of odds ratio relative to risk ratio^c^

1	Surviving Ebola virus [32]	Safe sexual behavior	14%	2.71	3.67	35%
2	Point-of-care testing [33]	Antibiotic use	78%	0.82	0.50	178%
3	Drinking [34]	Feelings of aggression	20%	3.1	6.7	116%

^a^ Risk ratio (for “exposed” vs. “unexposed”) computed directly from outcome proportions reported in the article as none of the three articles used the risk ratio as a measure of relative association. ^b^ Odds ratio is obtained from unadjusted logistic regression [32] or directly from outcome proportions reported [33,34]. ^c^ In these examples where the outcome is relatively common (i.e., >10%), if the odds ratio were to be incorrectly interpreted as a risk ratio, this is the magnitude of overstatement of relative association.

At this stage, it is valuable to make a note on the terminology of “risk” and “risk ratio”. Although in the strictest sense, a risk is defined in epidemiology as the “probability of an event during a specified period of time [9]”, in common usage, “risk” refers more generally to a probability, and the term risk ratio or relative risk is commonly used in research to describe a relative association, even when the probability does not involve an element of time (e.g., cross-sectional prevalence of an outcome). Hence, we use the term “risk ratio” (RR) throughout to refer to a ratio of probabilities or prevalences.

### Uses and (Mis)interpretations

Although the authors did not misinterpret the degree of association of the subsidy intervention and the proportion who tested for malaria, the O’Meara example can be used to demonstrate the potential for misinterpretation of the OR. We have stated that the RR of 1.45 can be interpreted as “receiving RDT subsidy is associated with being 1.45 times more likely to test for malaria compared to those not receiving a subsidy”. But, as Schwartz et al. [8] point out in critiquing a prominent study and the media’s interpretation of the results, this is *also* how the OR is commonly interpreted. It is natural to want to interpret the OR of 2.7 as “2.7 times more likely to test for malaria if receiving RDT subsidy.” However, this is *not* the correct interpretation. In the O’Meara et al. example, if the OR is incorrectly interpreted as an RR, it overstates the intervention effect by almost double (1.45*2 = 2.9).

As pointed out by many authors, the interpretation of an OR is not intuitive, and ORs are easily misinterpreted [7,10,11]. And, importantly, even when authors are careful in their interpretation, for instance by using language such as “receiving treatment is associated with 2.7 times the odds of outcome compared with control”, it is still natural for the news media and other readers of the research to interpret it as an RR—that is, a ratio of probabilities rather than a ratio of odds [8,9,10,11,12].

**Figure 1:**
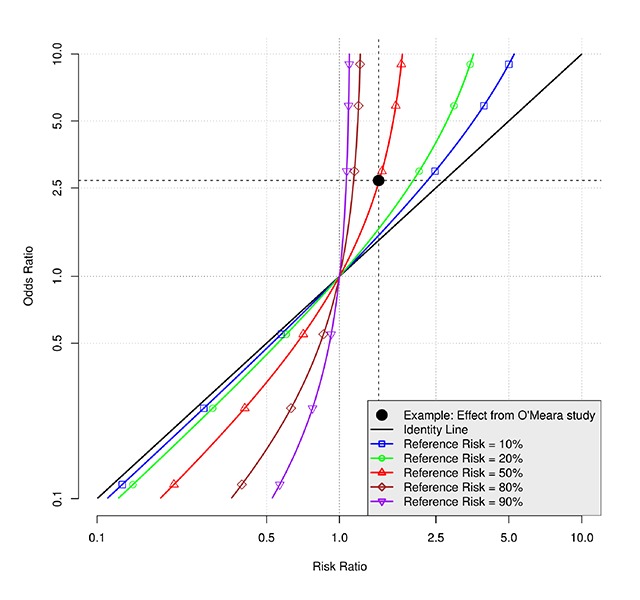
Relationship between the odds ratio and risk ratio at various levels of the reference risk.

In the example above, the OR and RR were so different because the proportion of the study sample testing for malaria (the outcome) was so high (51.0% in control). In the case of a high outcome proportion, the OR is pulled away from the null value (i.e. an OR and an RR of 1.0) more than the RR. In Figure 1, we display this relationship by graphing the OR and RR for various levels of the reference probability (e.g., probability of testing for malaria in the no subsidy arm), including for settings where the intervention is associated with a reduction in the probability of the outcome (i.e. with the RR and OR both <1). As can be seen, the higher the reference probability of the event (e.g., tested for malaria) the more the OR overstates the RR (i.e., OR < RR if both are <1 and OR > RR if both are >1), if the OR is incorrectly interpreted as an RR. Similarly, the figure shows that when the outcome is not common (e.g., <10%), the OR closely approximates the RR, and therefore, in such cases the OR may be interpreted as an RR.

### Why does this matter?

Although there is some literature on methods to compute RRs for binary outcomes, most of it has appeared in epidemiological [13,14,15,16,17,18,19], medical [11,20,21,22,23,24,25,26,27], or statistics journals [28,29,30,31]. This may partly explain why ORs are still commonly used and misinterpreted across a wide array of papers and throughout the media. The following three examples from the global health literature, and which are summarized in Table 2, present further evidence to the global health research community about why this issue is important.

In the first example, it was reported that Ebola virus disease survivors “were more than five times as likely to engage in safe sexual behavior compared with the comparison group”, based on an adjusted OR of 5.59, obtained from a logistic regression model with adjustment for relevant covariates selected by the authors [32]. Given that the corresponding unadjusted OR is 35% larger than the unadjusted RR (3.67 vs. 2.71), the adjusted RR is expected to be approximately 4.14 (vs. 5.59), which would indicate that survivors were “more than four times as likely to engage in safe sexual behavior”. This example shows that even in studies with a relatively low reference probability of the outcome of interest, interpreting the OR as an RR would lead to an overstatement of the association.

In a second example from the global health literature, an adjusted OR of 0.49 comparing antibiotic use in the intervention (64%) and control (78%) groups was estimated in a RCT of a point-of-care testing intervention to reduce antibiotic use [33] The unadjusted OR of 0.50 corresponds to an unadjusted RR of 0.82. If this OR were interpreted as an RR, the association of the intervention and outcome would be overstated almost three-fold (i.e., 50% vs. 18% reduction). This article can be used to further emphasize why the distinction between the two relative measures of association is important. Suppose that the intervention is associated with an increase in adverse events. Reporting the OR instead of the RR may make it more challenging to balance the pros of the intervention in terms of reduction in antibiotic overuse vs. the cons due to adverse events. The more high-impact the study, the more likely the conclusions will affect policy and, hence, will affect people’s lives.

The third example from the global health literature is an example of misinterpretation of the OR in the news media. The article reports on the results of a study examining the emotions associated with alcohol consumption [34], in which an adjusted OR of 6.41 comparing heavy drinkers with light drinkers for the outcome of “feelings of aggression” was interpreted as heavy drinkers were “just over six times more likely to report feelings of aggression” than light drinkers [34]. In reporting on the study the news article picked up on this interpretation and stated that “those who showed signs of alcohol dependence were six times more likely to say they felt aggression while drinking [12]”. However, using numbers provided in Table 2 of the paper we find an unadjusted OR for this association of 6.7 while the unadjusted RR is 3.1. Thus, the OR overstates the RR more than twofold and, instead, a more appropriate interpretation would be that “heavy drinkers were just over three times more likely to report feelings of aggression than light drinkers”.

## Methods of Obtaining Risk Ratios For Binary Outcomes

Given the difficulty in interpreting the odds ratio, several methods of obtaining risk ratios have been proposed in the literature and have been implemented in a range of studies. For simplicity and brevity, here we describe some advantages and disadvantages of four commonly used methods [29]. Summaries and examples of use of these four methods in the global health literature are given in Table 3. Two of the methods are regression-based approaches that directly estimate the RR (log-binomial [35] and modified log-Poisson [16]). In this case, the estimated coefficient of the association between exposure and outcome, when exponentiated, is directly interpreted as a risk ratio. The other two methods indirectly obtain the RR (substitution [25] and standardization [18]) by estimating an OR from a logistic regression model, then computing the RR as a function of the OR through some form of transformation. Although commonly used in many settings, these methods are only necessary when adjusting for covariates. This is because when there is no need to adjust for covariates, simple formulas can be used instead of modeling. Nevertheless, in many cases, researchers will use model-based methods even when not adjusting for covariates as they are similarly valid in unadjusted models and are straightforward to implement.

**Table 3:** Brief summary of four methods of obtaining risk ratios for binary outcomes.

Name of method	Type of method	Background literature	Some advantages	Some disadvantages	Example of use in the global health literature	Exposure	Binary Outcome

Log-binomial	Direct	Wacholder (1986) [35]	Easy to implement.	May not converge; may estimate individual-level probabilities (and/or the upper bound of their 95% confidence intervals) above 1.	Gibson et al. (2017) [37]	Mobile phone based intervention to improve immunization rates, in a cluster-randomized trial	Full immunization by 12 months of age.
Modified log-Poisson	Direct	Zou (2004) [16]	Easy to implement; almost always converges.	May estimate individual-level probabilities (and/or the upper bound of their 95% confidence intervals) above 1.	Chan et al. (2017) [38]	AIDS-related stigma	Probable depression (PHQ-9 score ≥10 or recent suicidal thoughts).
Substitution	Indirect	Zhang and Yu (1998) [25]	Easy to implement. Uses output from logistic regression.	Generally produces biased estimates and 95% confidence intervals are expected to be too narrow, on average [18].	Agweyu et al. (2018) [39]	Various demographics and health-related exposures	Mortality.
Marginal or Conditional Standardization	Indirect	Localio et al. (2007) [18]	Uses output from logistic regression.	May be more difficult to implement and interpret than other methods, especially in certain software packages.	Weobong et al. (2017) [40]	Psychological intervention for depression, in a randomized trial	Remission from depression as measured by the PHQ-9.

Abbreviation: PHQ-9 – Patient Health Questionnaire 9-item [36], a screening tool for depression.

The first direct estimation method, the log-binomial approach, is a generalized linear model like logistic regression which also uses a binomial outcome distribution but uses a log link rather than a logit link function [35]. While this is an attractive option because it is simple to implement in standard statistical software, the model may fail to converge to a solution, especially when the outcome is common [18,41].

The second direct estimation method is the modified log-Poisson model [16]. In this approach, a Poisson model with log-link is fitted to the binary data, which is “modified” by using robust standard errors to obtain valid statistical inference. This approach is simple to implement in many statistical software packages, and generally does not suffer from the same convergence issues as the log-binomial.

While the log-binomial and modified log-Poisson regression approaches are appealing, both may estimate individual-level outcome probabilities and/or the upper bound of their 95% confidence intervals above one for binary outcome data. If the intention is inference about associations, this is generally not a major issue. However, if the goal is estimation of individual-level risk, then these two methods will sometimes be inappropriate and estimate individual-level risk above one, especially when the outcome is very common and the variance of adjustment variables is high [11,42].

Two logistic-regression based approaches that indirectly obtain the RR through transformation are substitution and standardization. Zhang and Yu [25] proposed a substitution method, in which a simple formula—which includes the odds ratio and the prevalence of the outcome in the unexposed group—is used to convert the OR (and its 95% confidence interval [CI]) obtained using a standard logistic regression model to an RR (with 95% CI). Although this method is often cited and used in practice, simulations suggest that 95% CIs obtained using this method suffer from poor coverage, such that they are too narrow and type I error is inflated (too high) [18]. Additionally, several authors have pointed out that such simple substitution methods produce biased RRs [13,14,18,20]. Thus, this method is not recommended and there is currently no simple formula with desirable statistical properties to convert an OR to an RR.

The second indirect logistic regression-based approach is the standardization method proposed by Localio et al. [18] and described in further detail in Muller & MacLehose [43]. This method fits a logistic regression model and uses the estimated regression coefficients to obtain an estimated RR by using marginal standardization whereby the proportions with the outcome in the exposed and unexposed groups are estimated and, from these, the corresponding RRs are estimated. We obtain these proportions as the estimated probability of the outcome within each unexposed and exposed group at specified values of the other covariates in the model (e.g., at the mean of continuous variables). Under certain assumptions, this marginalized effect at each level of the exposure is the prevalence of the outcome we would have observed had everyone been assigned to that level of the exposure and to the specified values of the other covariates in the model. That is, although termed a “marginalized effect” when adjusted for covariates in this way, it is also conditional on the level of those covariates [43].

Cummings compares these methods with others and finds that, except for the often biased substitution method, they generally produce similar estimates of the RR [29]. Therefore, on balance, we find that the most easily implementable approach with the fewest drawbacks is the modified log-Poisson approach. Given that it is as easy to implement as logistic regression in all major software, there should be no barriers to global health researchers estimating and reporting the RR as a measure of relative association of an exposure and binary outcome, when study design allows. In Table 4, we provide code for fitting both the log-binomial and modified log-Poisson models in four commonly used statistical software (R, SAS, Stata, and SPSS), as well as code to implement the marginal standardization approach in both R and Stata. For the marginal standardization approach, SAS and SPSS code are not provided as to our knowledge it is not easily implemented in these two programs.

**Table 4:** Code to fit the log-binomial and modified log-Poisson models in four commonly used statistical software packages, and to use the marginal standardization method in two of the packages.

Software Program	Data Structure	Log-binomial code^a^	Modified log-Poisson code^b^	Marginal standardization code^c^

Stata^e^	Ind	glm binaryoutcome exposure, family(binomial) link(log) eform	glm binaryoutcome exposure, family(poisson) link(log) vce(robust) eform	logit binaryoutcome i.exposure, or margins exposure, coeflegend post nlcom (RR: _b[1.exposure]/_b[0bn.exposure] ), post
	Clust^d^	xtset cluster xtgee binaryoutcome exposure, family(binomial) link(log) corr(exchangeable) eform	xtset cluster xtgee binaryoutcome exposure, family(poisson) link(log) corr(exchangeable) eform	xtset cluster xtgee binaryoutcome i.exposure, family(binomial) link(logit) corr(exchangeable) eform margins exposure, post coeflegend nlcom (ratio1: _b[1.exposure]/_b[0bn.exposure] ), post
SAS	Ind	proc genmod data=temp; class binaryoutcome exposure / param=ref ref=first; model binaryoutcome = exposure / dist=bin link=log; estimate ‘Risk Ratio’ exposure 1 /exp; run;	proc genmod data=temp; class binaryoutcome exposure participantID / param=ref ref=first; model binaryoutcome = exposure / dist=poisson link=log; repeated subject=participantID / type=Ind; estimate ‘Risk Ratio’ exposure 1 / exp; run;	
	Clust	proc genmod data=temp descending; class binaryoutcome exposure cluster / desc; model binaryoutcome = exposure / dist=binomial link=log; repeated subject=cluster / corr=exch; estimate ‘Risk Ratio’ exposure 1 -1 /exp; run;	proc genmod data=temp descending; class binaryoutcome exposure cluster / desc; model binaryoutcome = exposure / dist=poisson link=log; repeated subject=cluster / corr=exch; estimate ‘Risk Ratio’ exposure 1 -1 /exp; run;	
R^f^	Ind	lglm <-glm(binaryoutcome~exposure, family=binomial(link=”log”)) exp(cbind(coef(lglm), confint(lglm)))	library(gee) pglm <-summary(gee(binaryoutcome~exposure, family=(poisson(link=”log”)),id=p articipantID,corstr=”independ ence”)) cbind(RiskRatio=exp(pglm$coefficients[2]), LCI=exp(pglm$coefficients[2]- 1.96*pglm$coefficients[8]), UCI=exp(pglm$coefficients[2]+1.96*p glm$coefficients[8]))	library(epitools) lglm <-glm(binaryoutcome~exposure, family=binomial) probratio(lglm,method=”ML”)
	Clust	library(gee) lgee<-summary(gee(bina ryoutcome~exposure, family=(binomial(link=”log”)), id= cluster,corstr=”exchangeable”)) cbind(RiskRatio=exp(lgee$coefficients[2]), LCI=exp(lgee$coefficients[2]- 1.96*lgee$coefficients[8]), UCI=exp(lgee$coefficients[2]+1.96*l gee$coefficients[8]))	library(gee) pgee<-summary(gee(binaryoutcome~exposure, family=(poisson(link=”log”)), id=cl uster,corstr=”exchangeable”)) cbind(RiskRatio=exp(pgee$coefficients[2]), LCI=exp(pgee$coefficients[2]- 1.96*pgee$coefficients[8]), UCI=exp(pgee$coefficients[2]+1.96*pg ee$coefficients[8]))	
SPSS	Ind	genlin binaryoutcome (reference=first) by exposure (order=descending) /model exposure intercept=yes distribution=binomial link=Log /Print summary solution(exponentiated)	genlin binaryoutcome (reference=first) by exposure (order=descending) /model exposure intercept=yes distribution=poisson link=log /repeated subject=participantID sort=yes corrtype=independent adjustcorr=yes covb=robust /Print summary solution(exponentiated)	
	Clust	genlin binaryoutcome (reference=first) by exposure (order=descending) /model exposure intercept=yes distribution=binomial link=Log /repeated subject=cluster sort=yes corrtype=exchangeable adjustcorr=yes covb=robust /Print summary solution(exponentiated)	genlin binaryoutcome (reference=first) by exposure (order=descending) /model exposure intercept=yes distribution=poisson link=Log /repeated subject=cluster sort=yes corrtype=exchangeable adjustcorr=yes covb=robust /Print summary solution(exponentiated)	

Abbreviations: Ind = Independent (i.e., non-clustered); Clust = Clustered. Variables: binaryoutcome = the binary outcome; exposure = exposure (e.g., treatment group indicator), assumed to be categorical; participantID = participant identifier; cluster = cluster identifier. ^a^ The log-binomial code for direct estimation of the risk ratio in the clustered setting is only shown in the generalized estimating equations (GEE) framework. A generalized linear mixed model (GLMM) could also be used. ^b^ For the log-Poisson approach, a robust standard error is needed to account for misspecification of the outcome distribution (i.e., Poisson instead of binomial); GEE is the natural approach to obtain this robust standard error, in both the non-clustered and clustered setting. ^c^ To our knowledge, the marginal standardization method is not as straightforward to implement in SAS or SPSS, so no code is provided. In addition, we are unaware of an easy-to-implement function in R to perform marginal standardization in a clustered setting. ^d^ In the context of GEE to analyze clustered outcome data, we have used an exchangeable working correlation matrix as an example. It is natural to use such a working correlation matrix when the outcome data are measured at a single point in time and the clustering arises through some natural grouping of individuals (e.g., in schools or hospitals). But, if the clustering arises from longitudinal data, other working correlation structures may be preferred. ^e^ The standard errors from Stata may be slightly larger than that obtained from the other programs. This is because Stata multiplies the robust standard errors by K/(K–1), where K is the number of clusters, whereas other programs do not do this. ^f^ The cbind R code illustrated here works only for a single binary exposure variable. It will need to be modified for more complex scenarios. Additionally, the gee function requires that the outcome be set up as a numeric variable, rather than a factor variable, when specifying the modified log-Poisson model.

Additionally, cluster randomized trials (CRTs) are common in global health research. In such cases, statistical models must take into account the fact that the data collected on participants within the same cluster are likely to be correlated. When the outcome is binary, the generalized estimating equations (GEE) approach is an appealing method to analyze CRT data because of its desirable statistical properties (e.g., population-average interpretation; robustness to model misspecification; ability to correct for small-sample bias in the case of fewer than 40 clusters enrolled in the CRT) [44,45,46]. Both the log-binomial and modified log-Poisson [30] models can be easily implemented in the GEE framework, and code to do so is provided in Table 4. Similarly, in some software, the marginal standardization procedure can also be easily adapted to clustered binary outcome data. This code could also be used for non-randomized studies with clustering of outcomes.

## Absolute Measures of Association

Just as RRs can be directly estimated as a measure of relative association, direct and indirect methods are available to estimate absolute measures of association such as risk differences. Although a discussion of the advantages and disadvantages of such methods is beyond the scope of the current article, it is important to note that absolute measures of association are able to provide important and complementary information about the public health impact of interventions. In fact, both the Consolidated Standards of Reporting Trials (CONSORT) and the Strengthening the Reporting of Observational Studies in Epidemiology (STROBE) statements recommend that all RCTs and observational studies reporting on the association of an exposure with binary outcomes provide both a relative measure and absolute measure [47,48].

As an example of the importance of reporting both types of measures, suppose that the probability of malaria testing uptake in the RDT subsidy arm was 0.02%, while in the no subsidy arm it was 0.01%. In this case, the risk ratio is 2, whereas the risk difference is 0.01 percentage points. From a public health perspective, such a small risk difference may be considered of insufficient magnitude to justify the increased costs and potential adverse effects of providing subsidies. However, if only the relative measure were reported, those involved in scaling up the intervention may be misled to believe the intervention is more effective than it actually is. Thus, we also strongly recommend reporting both a relative and absolute measure of association when reporting on binary outcomes.

The statistical methods for obtaining an absolute measure of association (specifically, the risk difference) are straightforward to implement. For both the modified log-Poisson and log-binomial models, instead of using a log link, an identity link can be used. In this case, the regression coefficients will have a straightforward interpretation as the difference in risk between the levels of the predictor (e.g., intervention and control). Additionally, for the marginal standardization method, once we have obtained the estimated mean risks for the levels of the predictor, we simply subtract the estimated mean risks to obtain the risk difference. In sum, the same methods that can be used to obtain risk ratio can also be used to obtain risk differences with only minor modifications.

## Conclusion

We have shown that many methods exist to estimate risk ratios for binary outcome data and that the global health researcher need not feel compelled to present odds ratios for studies with sampling which depends upon the exposure, such as cohort and cross-sectional observational studies and randomized controlled trials. Overall, the modified log-Poisson regression approach to generate RRs is generally preferred to alternative approaches due to its ease of implementation and desirable statistical properties. While we have not provided an exhaustive review of methods for estimating RRs, Cummings [20] provides an excellent review of alternative methods, and also provides Stata code for implementing various approaches [29]. In addition, Muller & MacLehose discuss marginal standardization (as well as other methods), and provide Stata code for implementing marginal standardization [43]. As noted earlier, sample code for fitting the two direct regression approaches in four statistical programs, as well as performing marginal standardization in two of those programs, is provided in Table 4. As with fitting any model, researchers should be aware of and test assumptions underlying the model, and consider how interpretation changes when adjusting for covariates.

Given concerns with interpretation, especially since results of research are commonly used to implement and scale up interventions, we believe that estimation and reporting of odds ratios should be reserved for use mainly when performing case-control studies. In this case, the risk ratio will be directly estimated by the OR if base population sampling (i.e., a case-cohort design) is used. It should be noted, however, that for other forms of control sampling (i.e., risk-set sampling and survivor sampling), the risk ratio would not be validly estimated by the odds ratio [49].

We have provided researchers with the information needed to decide upon the most appropriate and interpretable measures of relative association to present in studies with binary outcomes, while also describing in detail the tools needed to obtain these relative measures. Our hope is that this information will assist researchers in providing the best evidence on the association between exposures and binary outcomes in observational studies, as well as on the effectiveness of interventions evaluated in RCTs in global health settings.
