# Corrigendum: Relative Measures of Association for Binary Outcomes: Challenges and Recommendations for the Global Health Researcher

**DOI:** 10.5334/aogh.2998

**Published:** 2020-07-06

**Authors:** John A. Gallis, Elizabeth L. Turner

**Affiliations:** 1 Duke Global Health Institute, Duke University, Durham, North Carolina, US; 2 Department of Biostatistics and Bioinformatics, Duke University School of Medicine, Durham, North Carolina, US

## Abstract

This article details a correction to the article: Gallis, J. A. and Turner, E. L., 2019. Relative Measures of Association for Binary Outcomes: Challenges and Recommendations for the Global Health Researcher. *Annals of Global Health*, 85(1), p. 137. DOI: http://doi.org/10.5334/aogh.2581

## Corrigendum

Shortly after pubication of Gallis and Turner [1] the authors informed us that they accidentally omitted an important option in the Stata code of Table 4.

In the clustered setting, the generalized estimating equations (GEE) Stata code must include the option “vce(robust)” in order to obtain robust standard errors. In the modified log-Poisson model, this option is essential in order to obtain valid statistical inference. The “vce(robust)” option may also be added for the log-binomial model when clustering is present, and should be added in order to match the output from the R, SAS, and SPSS code, which report the robust standard errors.

The updated modified log-Poisson code in the clustered setting is:

xtgee binaryoutcome exposure, family(poisson) link(log) corr(exchangeable) vce(robust) eform

This omission does not affect any data published in the article.
